# Impact of Anastomotic Leakage After Colorectal Cancer Surgery on Quality of Life: A Systematic Review

**DOI:** 10.1097/DCR.0000000000003478

**Published:** 2024-10-23

**Authors:** Anke H.C. Gielen, Danique J.I. Heuvelings, Patricia Sylla, Yu-Ting van Loon, Jarno Melenhorst, Nicole D. Bouvy, Merel L. Kimman, Stephanie O. Breukink

**Affiliations:** 1 Department of Surgery, Maastricht University Medical Centre, Maastricht, The Netherlands; 2 School of Nutrition and Translational Research in Metabolism (NUTRIM), Maastricht University, Maastricht, The Netherlands; 3 Division of Colon and Rectal Surgery, Icahn School of Medicine at Mount Sinai, New York, New York; 4 GROW School for Oncology and Developmental Biology, Maastricht, The Netherlands; 5 Department of Clinical Epidemiology and Medical Technology Assessment (KEMTA), Maastricht University Medical Centre, Maastricht, The Netherlands

**Keywords:** Anastomotic leakage, Colorectal cancer surgery, Quality of life

## Abstract

**BACKGROUND:**

Colorectal anastomotic leakage remains one of the most frequent and dreaded postoperative complications after colorectal resection. However, limited research has been conducted on the impact of this complication on the quality of life of patients who have undergone colorectal cancer surgery.

**OBJECTIVE:**

The aim of this systematic review was to identify, appraise, and synthesize the available evidence regarding the quality of life in patients with anastomotic leakage after oncological colorectal resections to inform clinical decision-making.

**DATA SOURCES AND STUDY SELECTION:**

PubMed, Embase, and the Cochrane Library were searched for studies reporting quality of life using validated questionnaires in patients with anastomotic leakage after oncological colorectal resections. The literature search was performed systematically and according to Preferred Reporting Items for Systematic Reviews and Meta-Analyses guidelines.

**OUTCOMES:**

Outcomes from quality-of-life questionnaires of patients with and without anastomotic leakage were analyzed.

**RESULTS:**

Thirteen articles reporting on 4618 individual patients were included, among which 527 patients developed anastomotic leakage. Quality of life was evaluated using 10 distinct questionnaires administered at various postoperative time points, ranging from 1 month to 14 years. Quality-of-life outcomes differed across studies and time points, but overall scores were most negatively affected by anastomotic leakage up to 12 months postoperatively.

**LIMITATIONS:**

There was a high heterogeneity between the included studies based on the questionnaires used and the time of assessment.

**CONCLUSIONS:**

The published evidence suggests that anastomotic leakage after oncologic colorectal resection is associated with impaired quality of life, especially within the first postoperative year. The impact of anastomotic leakage on quality of life warrants further evaluation and discussion with patients.

Oncological colorectal resection with or without primary anastomosis remains the cornerstone in the treatment of colorectal cancer (CRC). In patients undergoing restorative procedures, anastomotic leakage (AL) remains one of the most frequent and dreaded postoperative complications, with a reported incidence varying from 1.5% to 20%.^1–4^ This wide-ranging incidence in the literature may be due to differences in surgical risk among different study populations and variability in surgical techniques, but it also reflects significant differences in reporting standards for AL. Although several classifications and definitions of AL have been described in the literature, there is no consensus on definitive diagnostic or clinical criteria for AL.^5–8^

Several important risk factors for AL have been identified during the past few decades, such as active smoking, malnutrition, male sex, obesity, emergency surgery, operative time, postoperative use of nonsteroidal anti-inflammatory drugs, and neoadjuvant chemotherapy.^9–11^ Despite innovations in surgical techniques, preoperative optimization, and intraoperative interventions to further minimize the risk of AL, rates of anastomotic complications have not decreased. AL ranges in clinical severity from minor, subclinical, and contained leaks to fulminant sepsis and organ failure with increased short-term mortality rates.^12^

A standardized consensus framework for defining, reporting, and grading colorectal AL is currently being developed by the Consensus on Reporting and Defining Colorectal Anastomotic Leaks. This expert group noticed gaps in knowledge about the short- and long-term impact AL on functional outcomes and overall quality of life (QoL). As patients should be fully informed not only regarding the immediate surgical risks but also on the impact surgical complications may have on long-term function and QoL, this systematic review was undertaken to address this important question about the short- and long-term impacts of AL in patients with CRC. The aim of this systematic review was to identify, appraise, and synthesize the available evidence regarding short- and long-term QoL in patients who have undergone oncological colorectal resections complicated by AL.

## MATERIALS AND METHODS

### Study Protocol and Registration

This systematic review was conducted according to the latest edition of the Preferred Reporting Items for Systematic Reviews and Meta-Analyses guidelines.^13^ The study protocol was developed a priori and registered at PROSPERO (ID 411065).

### Outcomes and Definitions

The primary outcomes were QoL and health-related QoL (HRQoL). QoL was defined using the World Health Organization definition of “an individuals’ perception of their position in life in the context of the culture and value systems in which they live and in relation to their goals, expectations, standards, and concerns.”^14^ HRQoL in cancer is often used interchangeably with QoL because there is no consensus on a standardized definition. We have applied the definition of Testa and Simonson^15^ on HRQoL as the “physical, psychological and social domains of health, seen as distinct areas that are influenced by a person’s experiences, beliefs, expectations and perceptions.”

AL was defined as a combination of clinical signs and symptoms (eg, abdominal pain or tenderness, peritonitis, fever, tachycardia, purulent or fecal discharge from an abdominal drain or the vagina, purulent discharge per anus), biochemical elements (elevated white blood cell count and/or C-reactive protein), and radiological confirmation of an interruption of the anastomosis and/or a perianastomotic collection on CT.^6,16^

### Search and Information Sources

The literature search was performed on March 13, 2023, and repeated on August 14, 2023. PubMed, Cochrane Library, and Embase were searched with the use of MeSH-, Emtree-, and free terms, including “colorectal neoplasms,” “(adeno)carcinoma,” “colorectal surgery,” “anastomotic leak,” “complications,” “quality of life (QoL),” and “health-related quality of life (HRQoL)” (see Supplemental S1 at http://links.lww.com/DCR/C410). Reference lists of relevant publications were cross-checked to identify additional studies. This hand-search method was continued until no further relevant studies were identified.

### Selection Process

#### Eligibility criteria and selection process

All English or Dutch articles published in peer-reviewed index journals reporting on QoL in patients older than 18 years with AL after oncological colorectal resections were considered eligible for inclusion. Analysis of QoL after AL had to be identified as a predetermined aim in the Methods section of the study to be eligible for inclusion. Trials were included irrespective of blinding.

Systematic reviews and secondary sources such as letters to the editor, technical descriptions, conference proceedings, and commentaries were excluded. Articles reporting on fewer than 10 patients or solely reporting on outcomes after colorectal resections for benign indications were excluded. Since the first systematic review on the definition of AL was published in the year 2001, all articles published before January 1, 2000, were excluded.^8^ Furthermore, articles were excluded when no validated HRQoL instrument had been applied.

All search results were imported into an online tool designed for systematic reviews (Rayyan).^17^ After the removal of duplicates, articles were screened for eligibility by 2 independent researchers (A.H.C.G. and D.J.I.H.) according to the predefined criteria. First, articles were screened on the basis of titles and abstracts. Definitive article inclusion followed if the eligibility criteria were met after full-text screening by both reviewers. Disagreements were resolved through discussion. All references were stored in the Endnote Reference Management Tool (version 20.4, Clarivate, Chandler, United States).

### Data Extraction and Synthesis of Results

Two independent researchers (A.H.C.G. and D.J.I.H.) performed a qualitative analysis and extracted data from the main text, tables, and figures using a predefined and standardized data extraction table. Extracted data included first author, year of publication, country, study design, study period, inclusion and exclusion criteria, aim of the study, number of patients, general patient characteristics, indication for surgery, surgical procedures performed, the applied (validated) QoL questionnaires, time of assessment, and secondary outcomes. Furthermore, definitions, time frame, and criteria for diagnosis of AL were collected. Data acquired via the outlined search strategy were summarized in tables. Findings were described using a narrative approach (ie, primarily words and text were used to summarize and explain the findings). Because of the heterogeneity among included studies in terms of the definition of AL and questionnaires used to assess QoL, pooling in a meta-analysis was impossible.

### Assessment of Risk of Bias in Individual Studies

To ascertain the validity of the included studies, the risk of bias in each study was assessed by 2 reviewers (A.H.C.G. and D.J.I.H.) with a revised Risk of Bias in Non-randomized Studies of Interventions tool to assess the risk of bias in nonrandomized studies.^18^ All types of bias were evaluated for every study and judged to be “low risk,” “moderate risk,” or “high risk.” Possible confounding domains were a priori defined as active smoking, malnutrition, male sex, BMI, comorbidities or higher ASA classification, emergency surgery, and longer operative time.

## RESULTS

### Study Selection

**FIGURE 1.**
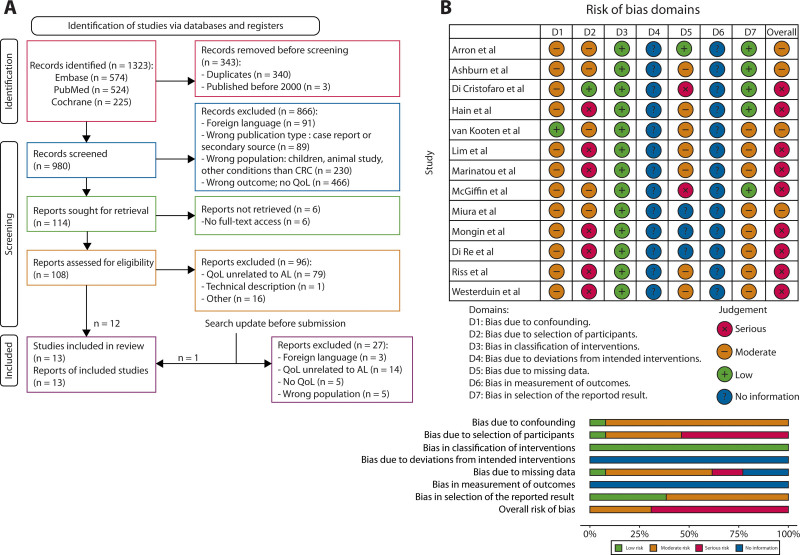
Study selection. A, PRISMA flow chart outlining study selection strategy. B, Adapted ROBINS-I tool Risk of Bias from included studies. CRC = colorectal cancer; PRISMA = Preferred Reporting Items for Systematic Reviews and Meta-Analyses; QoL = quality of life; ROBINS-I = Risk of Bias in Non-randomized Studies of Interventions.

The electronic literature search generated 1323 articles and 980 unique articles after removing duplicates. Of these, 866 were excluded after title and abstract screening. A full-text screening of the resulting 114 articles was performed and another 101 were excluded. Cross-reference checking generated 1 additional article, and 1 more additional publication was identified after repeating the search before submission. Ultimately, 13 articles were included in the analysis (Fig. 1A).

### Study and Patient Characteristics

All 13 articles included were cohort or case-matched studies and comprised 4618 individual patients, with study sample sizes ranging from 32 to 1207 patients (Table 1).^19–26,28–31^ Four studies reported on colorectal resections,^19,21,24,29^ and all other studies focused on rectal resections. All studies included patients diagnosed with CRC, with only 2 studies also including patients with benign indications for colorectal resections (eg, diverticulitis, IBD). Because benign indications were presented separately, the outcomes of these patients were excluded from this review.^24,29^ The final population consisted of 4618 patients, of whom 2946 (64%) were men and 1672 (36%) women, with a mean age of 61.9 years. Among these patients, 527 (11%) developed AL and 4091 (89%) recovered without clinical, radiological, or biochemical signs of AL (Table 2).^19–26,28–31^ The median time of follow-up was 4.3 years (4.8 months–14.4 years). Additional study information on the perioperative care of patients in each study is provided in Supplemental S2 at http://links.lww.com/DCR/C410.

**TABLE 1.** Characteristics of included studies

*Reference*	*Year*	*Country*	*Study design*	*Study* *period*	*No. of* *patients*	*Inclusion criteria*	*Exclusion criteria*	*Aim of the study*
Arron et al^19^	2023	The Netherlands	Observational cohort study	2010–2019	1197	Patients aged 18 y or older, diagnosed with stage I–III CRC	History of CRC, IBD, hereditary CRC syndromes, non-Dutch speaking or diagnosed with a mental condition that limited the ability to fill out questionnaires, nonelective surgery, resection without primary anastomosis, or with end stoma reconstruction	To assess whether AL is associated with HRQoL at 6 mo and 2 y postdiagnosis and whether AL is associated with a clinically relevant decrease in HRQoL at 6 mo and 2 y postdiagnosis compared to at the time of diagnosis
Ashburn et al^20^	2013	United States of America	Retrospective cohort study	1980–2010	864	Restorative resection for rectal cancers for tumors <15 cm from the anal verge	Patients with IBD, familial adenomatous polyposis, and hereditary and nonpolyposis colon cancer; patients undergoing nonrestorative resections	To evaluate the impact of AL, when intestinal continuity can still be maintained, on bowel function and QoL in patients undergoing rectal cancer resection with low colorectal or coloanal anastomoses
Di Cristofaro et al^21^	2014	Italy	Prospective cohort study	2020–2011	116	Patients admitted for elective CRC surgery	Emergency surgery, explorative laparotomy, inoperable CRC, or recurrence at follow-up	To investigate how postoperative complications after surgery for CRC affect patients’ QoL and satisfaction with care
Hain et al^22^	2017	France	Case-matched study	2005–2014	135	All patients undergoing laparoscopic sphincter-saving partial or total mesorectal excision	Patients with temporary or permanent stoma, no minimum follow-up of 1 y, and no current chemotherapy, and patients who were included in a previous study from the group that evaluated a postoperative program	To assess long-term functional outcomes after laparoscopic, sphincter-preserving operative intervention for rectal cancer according to the type of AL
van Kooten et al^23^	2022	The Netherlands	Observational cohort study	1996–1999	1207	Clinically resectable adenocarcinoma with an inferior tumor margin below the level of S1/S2 and within 15 cm from the anal verge without evidence of distant metastasis	Not having filled out the baseline HRQoL questionnaire, deceased within 30 d after surgery	To objectify the difference in short- and long-term HRQoL between uncomplicated and complicated postoperative recovery after TME for rectal cancer
Lim et al^24^	2006	United Kingdom	Observational cohort study	2000–2014	138	All patients undergoing surgical procedures with low rectal anastomosis (<10 cm from the anal verge).	NA	To evaluate the effect of (sub)clinical AL on QoL and the significance of features seen on water-soluble contrast enemas in the prediction of subsequent anastomotic healing
Marinatou et al^25^	2014	Greece	Retrospective case-matched study	2007–2012	75	Biopsy-proven CRC	Age <18 y or >80 y, distant metastatic disease at presentation, initially managed on an emergency basis, history of IBD or hereditary cancer	To explore the effect of clinically evident AL on HRQoL
McGiffin et al^26^	2022	Australia	Retrospective cross-sectional study	2004–2018	224	Patients undergoing minimally invasive proctectomy with a low extraperitoneal anastomosis and without a temporary diverting ileostomy	Age <18 y and tinability to give consent for survey participation, patients with temporary diverting ileostomy	To analyze the timing and management of AL and to evaluate the effect on long-term QoL and functional outcomes using validated instruments
Miura et al^27^	2018	Japan	Retrospective cohort study	2000–2012	275	Low rectal adenocarcinoma	Diverting stoma	To evaluate permanent stoma formation and defecation in long-term follow-up after surgery for low rectal cancer without a diverting stoma
Mongin et al^28^	2014	France	Retrospective case-matched study	2004–2010	63	Laparoscopic TME for rectal cancer with a follow-up of ≥6 mo and consent to fill in the questionnaires	Permanent or temporary stoma	To assess the influence of AL on long-term functional results and QoL after laparoscopic TME for cancer
Di Re et al^29^	2023	Australia	Retrospective case-matched study	2010–2020	122	AL, adult population, benign or malignant colorectal disease	IBD, pouch formation, and redo resections	To assess bowel function and QoL after AL from rectal resections
Riss et al^30^	2011	Austria	Retrospective case-matched study	1995–2006	32	TME for rectal cancer; Control group: uneventful postoperative course	Died during follow-up and had no response to questionnaire	To investigate the impact of AL after rectal resection for malignancies on overall pelvic organ function and QoL
Westerduin et al^31^	2021	The Netherlands, Belgium, and France	Retrospective comparative cohort study	2007–2017	170	TME for rectal cancer, all indications for redo anastomosis; Control group: successful primary anastomosis	Partial mesorectal excision, stoma at the time of questionnaire, inability to read or understand the questionnaires	To compare functional outcomes and the QoL between redo anastomosis and primary successful anastomosis after TME for rectal cancer

AL *=* anastomotic leakage; CRC *=* colorectal cancer; HRQoL *=* health-related quality of life; NA = not applicable; TME *=* total mesorectal excision.

**TABLE 2.** Patient characteristics

*Reference*	*Indication for surgery*	*Type of surgery*	*AL rate,* *yes: no* (%)	*Sex* *(M: F*)		*Age, y, mean ± SD or median (IQR*)		*Comorbidities/ASA*			*Clavien-Dindo*
				*AL*	*No AL*	*AL*	*No AL*	*ASA*	*AL*	*No AL*	
Arron et al^19^	Colon cancer (n = 895) Rectal cancer (n = 302)	Open and laparoscopic ileocecal resection, right and left hemicolectomy, transverse resection, sigmoid resection, subtotal colectomy, and LAR	63: 1134 (5.26%)	45: 18	706: 428	64.8 (59.7 to 69.7)	66.6 (61.7 to 71.6)	I II III+ Unknown	20 32 10 1	343 635 122 35	NA
Ashburn et al^20^	Rectal cancer	Any open or laparoscopic restorative rectal resection	52: 812 (6.02%)	43: 9	519: 293	56.9 ± NA	61.3 ± NA	I II III IV V	232 1910 26 1 0	8 395 369 18 0	NA
Di Cristofaro et al^21^	Colon cancer (n = 82) Rectal cancer (n = 34)	Any open and laparoscopic colorectal resection	5: 111 (4.31%)	71: 45		69.5 (61–75)		NA			Grade I: 3 Grade II: 17 Grade III: 9 Grade IV: 1
Hain et al^22^	Rectal cancer	Laparoscopic sphincter-saving PME or TME according to the principles of extrafascial dissection	46: 89 (34%)^a^	35: 11	65: 24	60.9 ± 8.3	63.5 ± 9.4	I II III	13 29 3	22 62 3	NA
van Kooten et al^23^	Rectal cancer	LAR, APR, and Hartmann resection	79: 1128 (6.55%)	767: 440		A: 64 (23–88) B: 65 (41–86) C: 66 (26–92) D: 67 (43–88)^b^		NA			NA
Lim et al^24^	Rectal cancer (n = 126), adenoma (n = 4), endometriosis (n = 2), Crohn's disease (n = 1), diverticulitis (n = 3) or postendomucosal resection (n = 2)	TME with anastomotic distance of ≤10 cm from the anal verge	23: 115 (16.67%)	11: 12	72: 43	Clinical 66 (54–81)	Subclinical 62 (51–75)	II III IV	Clinical AL 6 5 2	Subclinical 4 5 1	NA
Marinatou et al^25^	Rectal cancer	TME for lower and mid rectum tumors, and PME with transection of the mesorectum at least 5 cm distal to the tumor for upper rectum tumors	25: 50 (33.33%)^a^	15: 10	30: 20	62 ± 15.2	61 ± 16.3	I II III IV	1 9 13 2	5 20 23 2	NA
McGiffin et al^26^	Rectal cancer	Laparoscopic and robotic LAR with an extraperitoneal anastomosis	24: 200 (10.71%)	15: 9	119: 81	62 (52–69.8)	65 (56.3–73)	I II III–V	9 14 1	56 106 32	Used but not specified
Miura et al^27^	Rectal cancer	LAR (n = 157) and ISR (n = 118)	60: 215 (21.81%)	199: 76		64		ASA III–IV 30			Grade I/II: 62 Grade III: 66 Grade IV: 6 Grade V: 1
Mongin et al^28^	Rectal cancer	Laparoscopic sphincter-saving TME	21: 42 (33.33%)^a^	4: 17	11: 31	61 ± 9	60 ± 11	NA			NA
Di Re et al^29^	Colon cancer (n = 16), rectal cancer (n = 55), benign (n = 28)^c^	Laparoscopically, open, robotically, or with conversion LAR (n = 25), ultralow LAR (n = 50), and high anterior resection (n = 25)	61: 61 (50%)^a^	43: 18	41: 20	62.4 ± 12.3	64.1 ± 8.6	Cardiovascular disease n = 14 Diabetes mellitus n = 24 Renal failure n = 4 Lung disease n = 10 Liver disease n = 7			Grade I: 0 Grade II: 39 Grade IIIa: 49 Grade IIIb: 32 Grade IV: NA Grade V: 0
Riss et al^30^	Rectal cancer	ISR, LAR, and APR	16: 16 (50%)^a^	11: 5	11: 5	67 ± 11.2	70 ± 8.4	NA			NA
Westerduin et al^31^	Rectal cancer	Open and laparoscopic TME with anastomosis within 3 cm from the dentate line	52: 118 (30.59%)^a^	34: 18	79: 39	63 ± 8.9	68 ± 9.9	NA			NA

AL = anastomotic leakage; APR = abdominoperineal resection; IQR = interquartile range; ISR = intersphincteric rectal resection; LAR = low anterior resection; NA = not applicable; PME = partial mesorectal excision; TME = total mesorectal excision.

a AL rates do not reflect the incidence of AL due to the study design.

b Group A: no complications, group B: surgical complications, group C: nonsurgical complications, and group D: surgical and nonsurgical complications.

c Benign indications for resection were irresectable polyps or diverticulitis.

### Risk of Bias in Studies

The relevant categories from the Risk of Bias in Non-randomized Studies of Interventions tool were used to assess the risk of bias (Fig. 1B). We reported a serious risk of bias in 9 studies, primarily attributed to the nonrandomized design of these studies^21,22,24–26,28–31^ and a moderate risk of bias in the other 4 studies.^19,20,23,27^

### AL Characteristics

All details on AL and specific characteristics reported by each study are summarized in Supplemental S3 at http://links.lww.com/DCR/C410. The reported definitions and diagnostic modalities for AL varied widely among the studies reviewed. Four studies (33%) did not report any specific definition for AL.^21,28,30,32^ Furthermore, none of the studies applied the same definition for AL. The severity of AL was assessed using various classifications across the included studies. Four studies applied the International Study Group of Rectal Cancer classification,^19,26,27,30^ whereas 2 studies used the Clavien-Dindo classification.^21,25^ Four studies divided AL cases into symptomatic and asymptomatic or clinical and subclinical manifestations.^22,24,28,29^ The other studies did not provide a specific classification or grading of severity of AL. The time frame in which AL was suspected or diagnosed was reported in 4 articles, with the latest reporting time being 6 months after surgery.^19,22,24,27^ One study reported biochemical characteristics that might indicate surgical complications.^24^ Eight studies (67%) used CT with or without contrast to confirm the diagnosis of AL.^20,22–25,27–29^ Four studies reported performing radiological assessment and subsequent AL assessment only when clinical symptoms occurred.^19,20,25,27^ Three other studies additionally performed routine scanning for AL before ileostomy closure (range, 6 weeks–3 months after surgery).^22,24,28^ The type of reinterventions was specified in 10 studies^19,21,22,24–30^ and ranged from antibiotic treatment to reoperation (laparotomy) with take down of anastomosis and end-colostomy construction.

### Questionnaires

A total of 10 validated QoL questionnaires were administered at different time points within the studies. Four validated instruments were administered across the majority of studies (see Supplemental S4 at http://links.lww.com/DCR/C410: The European Organization for Research and Treatment of Cancer QoL Questionnaire [EORTC QLQ]-C30 [Core] and -CR29 [CRC-specific], the 36-item Short-Form survey [SF-36] encompassing both a physical component summary [PCS] and a mental component summary [MCS], and the Fecal Incontinence QoL [FIQL] questionnaire). Six additional questionnaires were used in only 1 study (Supplemental S5 at http://links.lww.com/DCR/C410). These included the Cleveland Global QoL (CGQL), the EORTC IN-PATSAT32 questionnaire for assessing cancer care satisfaction, the GI QoL Index (GIQLI) addressing digestive disorders with both physical and emotional components, the EuroQoL Visual Analog Scale for patient self-rated health, the 12-item Short-Form survey evaluating health impact on daily life, and the Rotterdam Symptoms Check List (RSCL) questionnaire, which generally evaluates HRQoL.

### QoL Scores

**FIGURE 2.**
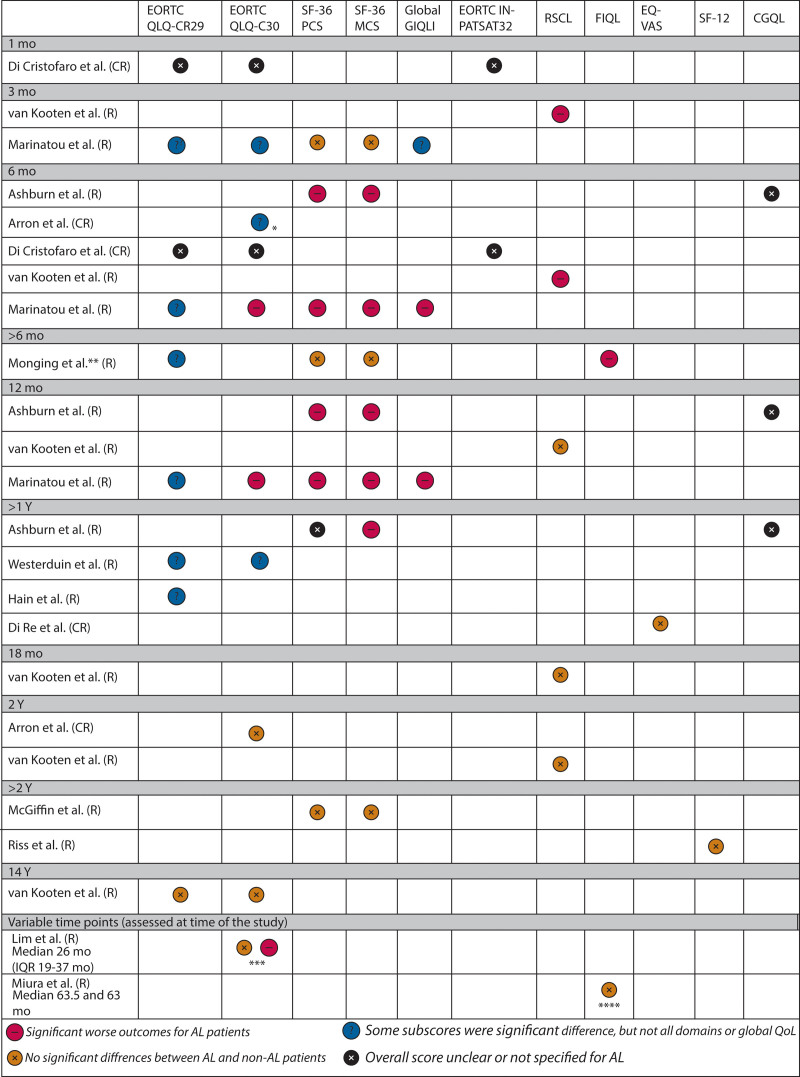
Schematic overview of results of QOL questionnaires based on different time points. *Not statistically significant but on an individual level AL was a determinant of a clinically relevant decrease; **at least 6 mo after the restoration of bowel continuity; ***subgroup: in patients with no stoma closure, there was a difference; ****modified version of the questionnaire. AL = anastomotic leakage; CGQL = Cleveland Global QoL questionnaire; EORTC = European Organization for Research and Treatment of Cancer; EQ-VAS = the EuroQoL Visual Analog Scale; FIQL = Fecal Incontinence; GIQLI = GI QoL Index; IQR = interquartile range; IN-PATSAT32 = inpatient satisfaction; MCS = mental component summary; PCS = physical component summary; QLQ-CR29 = QoL Questionnaire colorectal cancer–specific; QLQ-CR30 = QoL Questionnaire cancer–specific; QoL= quality of life; SF-12 = 12-item Short-Form survey; SF-36 = 36-item Short-Form survey; RSCL = Rotterdam Symptoms Check List.

QoL was evaluated at different time points. Almost all studies compared QoL scores between patients with AL and patients without AL at specific time points but not always relative to baseline assessment (Fig. 2; Table 3).

**TABLE 3.** Detailed overview of questionnaires and impaired domains if applicable

*Reference*	*Time of assessment after surgery*	*Questionnaire*	*Significant impaired domains (in favor of patients without AL patient*)	*p*	*Groups compared*
Arron et al^19^^a^	6 mo	EORTC QLQ-C30	Summary score^b^ Role function Social function Global QoL^b^ Physical function^b^ Emotional function^b^	0.00 0.00 0.00 0.00 0.01 0.01	AL vs non-AL after colorectal surgery at time of assessment and compared to baseline
			Multivariable linear regression analysis	Not significant	
	2 y	EORTC QLQ-C30	No significant outcomes	–	
			Multivariable linear regression analysis	Not significant	
Ashburn et al^20^^c^	6 mo and 1 y	SF-36 PCS	NA	0.01	AL vs non-AL after restorative proctectomy at time of assessment (not compared to baseline)
		SF-36 MCS	NA	0.007	
		CGQL	Unclear	Unclear	
	>1 y (range, 1–6 y)	SF-36 PCS	NA	Not significant	
		SF-36 MCS	NA	0.02	
		CGQL	Unclear	Unclear	
Di Cristofaro et al^21^^d^^,^^e^^,^^f^	1 mo	EORTC QLQ-CR29 EORTC QLQ-C30 EORTC IN-PATSAT32	Outcomes of questionnaires not specified for patients with AL		Patients without complications vs with complications at time of assessment (not compared to baseline)
			Multivariate analysis for AL: β = 0.42	0.01	
	6 mo	EORTC QLQ-CR29 EORTC QLQ-C30 EORTC IN-PATSAT32	Outcomes of questionnaires not specified for patients with AL		
			Multivariate analysis for AL: β = 0.52	0.004	
Hain et al^22^^g^	Variable, at time of the study (ranging from 1 to 10 y; mean 46 mo ± 26 after restoration of bowel continuity)	EORTC QLQ-CR29	Blood and mucus in stool Frequent bowel movements per day Frequent urination per day	0.045 0.04 0.03	Patients with symptomatic AL vs nonsymptomatic AL and patients without AL after rectal surgery at time of assessment (no baseline assessment performed)
van Kooten et al^23^^g^	3, 6, 12, 18, and 24 mo	RSCL	Global health Activity level	.01 0.01	AL vs non-AL after rectal surgery compared to baseline
	14 y	EORTC QLQ-C30	NA	Not significant	
		EORTC QLQ-CR29	NA	Not significant	
Lim et al^24^^d^^,^^e^	Variable, at time of the study (median 26 mo; IQR, 19–37 mo)	EORTC QLQ-C30	NA (only global score given)	0.03	Clinical leaks without stoma closure compared to subclinical leaks, clinical leaks with stoma closure and patients without AL after rectal surgery at time of assessment (no baseline assessment performed)
Marinatou et al^25^^d^	3 mo	EORTC QLQ-C30	Physical function	0.008	AL vs non-AL after rectal surgery at time of assessment (not compared to baseline)
		GIQLI	Physical function	0.03	
		EORTC QLQ-CR29	Abdominal and pelvic pain Stoma-related problems Sore skin	0.03 0.03 0.04	
		SF-36	NA	Not significant	
	6 mo	EORTC QLQ-C30	Physical function Global health status/QoL	0.03 0.002	
		GIQLI	Emotional function Physical function Global score	0.008 0.004 0.01	
		EORTC QLQ-CR29	Stoma- related problems Sore skin	0.002 0.03	
		SF-36	Role limitations due to physical health Role limitations due to emotional problems Social functioning General health	0.01 0.02 0.008 0.03	
	12 mo	EORTC QLQ-C30	Global health status/QoL	0.004	
		GIQLI	Emotional function Physical function Global score	0.007 0.03 0.005	
		EORTC QLQ-CR29	Sore skin	0.005	
		SF-36	Physical functioning Role limitations due to physical health Role limitations due to emotional problems Social functioning General health	0.04 0.001 0.003 0.009 0.002	
McGiffin et al^26^^d^	>2 y (median 6.4 y [IQR, 3.1–8.6]) and 4 y [IQR, 2.7–8.5] for no AL and AL group, respectively)	SF-36 PCS	NA	Not significant	AL vs non-AL after rectal surgery at time of assessment (no baseline assessment)
		SF-36 MCS	NA	Not significant	
Miura et al^27^^d^	Variable, at time of the study (median 63.5 and 63 mo for no AL and AL group, respectively)	modified FIQL	NA	Not significant	AL vs non-AL after rectal surgery at time of assessment (no baseline assessment)
Mongin et al^28^^d^	>6 mo after restoration of bowel continuity (median 33 mo [IQR, 6–75] and 30 mo [IQR, 6–70] for no AL and AL group, respectively)	SF-36 PCS	NA	Not significant	AL vs non-AL after rectal surgery at time of assessment (no baseline assessment)
		SF-36 MCS	NA	Not significant	
Di Re et al^29^^h^	Variable, at time of the study (range, 1–>5 y)	EQ-VAS	NA	Not significant	AL vs non-AL after colorectal surgery at time of assessment (no baseline assessment)
Riss et al^30^^i^	Variable, at time of the study (median 106.8 mo [range, 32.4–170.4])	SF-12	NA	Not significant	AL vs non-AL after rectal surgery at time of assessment (no baseline assessment)
Westerduin et al^31^^d^	>1 y (median of 41 mo [IQR, 23–71] and 27 mo [IQR, 16–43] for no AL and AL group, respectively)	EORTC QLQ-C30	Role function Social function Overall global health Fatigue Pain	0.049 0.006 0.002 0.04 0.002	AL vs non-AL after rectal surgery at time of assessment (no baseline assessment)
		EORTC QLQ-CR29	Body image Anxiety Abdominal pain Buttock pain Flatulence Fecal incontinence Sore skin	0.03 0.02 0.03 0.005 0.008 0.03 0.0007	

AL = anastomotic leakage; CGQL = Cleveland Global QoL; EORTC = European Organization for Research and Treatment of Cancer; EQ-VAS = the EuroQoL Visual Analog Scale; FIQL = Fecal Incontinence QoL; FISI = Fecal Incontinence Severity Index; GIQLI = Gastrointestinal QoL Index; IN-PATSAT32 = inpatient satisfaction; IQR = interquartile range; MCS = mental component score; NA = not applicable; PCS = physical component score; QLQ-CR29 = QLQ colorectal cancer–specific; QLQ-CR30 = QoL Questionnaire cancer-specific; QoL = quality of life; RSCL = Rotterdam Symptoms Check List; SF-12 = 12-item Short-Form survey; SF-36 = 36-item Short-Form survey.

a Multivariable linear and logistic regression.

b Not clinically relevant.

c Wilcoxon rank sum tests.

d Mann-Whitney *U* test.

e Kruskal–Wallis ANOVA.

f Multivariate analysis.

g Univariate and multivariate logistic regression.

h Wald’s tests using linear mixed-effects model and univariable Poisson regression analysis; Paired samples *t* test.

i Statistical analysis unclear.

#### QoL up to 6 months after surgery

Based on EORTC QLQ-CR29 and -CR30 scores at 1 and 6 months postoperatively, Di Cristofaro et al identified AL as an independent predictor of lower QoL in multivariate analysis (*p* < 0.001 and *p* = 0.004, respectively).^21^ van Kooten et al^23^ found that patients who developed AL reported a decrease in RSCL global health status and activity level within the first 3 months compared to preoperative scores, with some improvement at 6 months. In contrast, Marinatou et al^25^ did not document any improvement based on GIQLI and EORTC QLQ-C30 questionnaires administered at 3 and 6 months. Instead, a significant decline in physical functioning and global and overall QoL scores was documented among patients with AL relative to patients without AL at 6 months.^25^ Additional results from EORTC QLQ-C29 demonstrated significantly worse scores with respect to pain, stoma, and perianal skin-related complaints at 3 and 6 months in patients with AL. Also, SF-36 scores demonstrated significantly worse function among patients with AL versus patients without AL at 6 months, especially along emotional and social domains, which was not seen at 3 months. Impairments in functional outcomes based on SF-36 scores were also reported by Ashburn et al^20^ among patients with AL compared to patients without AL after proctectomy. Arron et al^19^ demonstrated that the decrease in EORTC QLQ-C30 scores observed among patients with AL at 6 months relative to patients without AL did not meet the threshold for clinical relevance, and AL status was not associated with the observed decrease. Among patients with a clinically relevant decrease in their 6-month scores relative to baseline, AL was an independent predictor of this decrease based on multivariate regression analysis.^19^

#### QoL at 12 months after surgery

Three studies reported QoL at 1 year after CRC resection.^20,23,25^ van Kooten et al^23^ demonstrated that HRQoL scores returned to baseline preoperative levels among rectal cancer patients with and without complications, whereas Marinatou et al^25^ demonstrated persistently significant differences between AL and non-AL groups for perianal skin soreness and worse overall EORTC QLQ-C30, global GIQLI, and SF-36 scores. Ashburn et al^20^ also documented significantly worse SF-36 scores in both the PCS and MCS domains at 1 year postoperatively in patients with AL compared to those without AL after restorative proctectomy.

#### Beyond 1 year after surgery

Mongin et al^28^ evaluated QoL in patients undergoing restoration of bowel continuity at least 6 months before the assessment. Given that the median time of QoL assessment was 33 versus 30 months in patients with versus without AL, results were interpreted as representing longer-term QoL. No difference in SF-36 scores were found between the 2 groups. However, “blood and mucus in stool” scores of the EORTC QLQ-CR29 indicated significantly worse function in patients with AL, as did depression/self-perception FIQL scores. Ashburn et al noted that although the SF-36 PCS scores did not show significant differences beyond 12 months postoperatively (median, 3.2 years), MCS scores were still significantly worse in patients with AL after proctectomy.^20^ Westerduin et al^31^ identified 5 domains of the EORTC QLQ-30 and 2 functional and 5 emotional domains of the -CR29, which were significantly better beyond 1 year postoperatively in patients with AL compared to patients without AL. Hain et al^22^ reported additional impaired -CR29 outcomes (more blood and mucus in stool, frequent bowel movements, and frequent urination per day) in patients with symptomatic AL compared to the combined groups of patients with no or asymptomatic AL. Di Re et al^29^ also demonstrated lower mean EuroQoL visual analog scale scores among patients with AL versus patients without AL in a matched cohort at 1 year after surgery (range up to 5 years), although the difference did not reach statistical significance.

At 18 and 24 months postoperatively, van Kooten et al found no differences in RSCL scores between patients with AL and patients without AL.^23^ Arron et al^19^ found no difference in overall HRQoL scores between patients with AL and patients without AL at 2 years relative to baseline EORTC QLQ-C30 scores.^19^ Similar results were described when SF-36 scores were compared >2 years after surgery between patients with AL (median of 4 years postoperatively) and without AL (median of 6.4 years postoperatively).^26^ Riss et al^30^ described no significant difference in mental and physical QoL scores measured by the 12-item Short-Form survey at a median follow-up time of 106.8 months after rectal surgery (range of 32.5–170.4 months) comparing AL to no AL patients from a matched cohort.

Two additional studies evaluated the longer-term impact of AL on QoL.^24,27^ Lim et al^24^ assessed the EORTC QLQ-C30 in patients without AL, with subclinical leaks, and with clinical leaks with and without ileostomy closure (overall median follow-up time of 26 months; interquartile range, 19–37 months). They found worse scores in patients with clinical leaks in whom ileostomy reversal was not possible. Miura et al^27^ did not find significant differences in overall modified FIQL scores when comparing patients with AL and patients without AL at a median time of 63 months after low rectal cancer surgery.

van Kooten et al^23^ conducted a supplementary analysis on EORTC QLQ-C30 and -CR29 outcomes 14 years postsurgery but found statistically significant differences between patients with AL and patients without AL.^23^

### Other Outcomes Related to QoL

Some additional outcomes that might influence QoL are summarized in Supplemental S6 at http://links.lww.com/DCR/C410. Neoadjuvant treatments were described by 9 studies,^19,20,22,24,25,28–31^ 1 of which found chemoradiation to be significantly different between patients with AL and patients without AL and 1 of which found radiotherapy to be significantly different.^25,31^ Diverting stoma rates between patients with AL and patients without AL were compared in 6 studies,^19,20,25,29–31^ of which 2 found significant differences (more diverting in the AL group).^20,30^ Stoma status during follow-up was clearly described by 2 studies,^19,25^ which showed significant differences between patients with AL and patients without AL within the first year after surgery. Two additional studies described permanent stoma rates related to AL.^24,27^ Di Re et al^29^ additionally analyzed oncological outcomes as disease-free survival at 1, 3, and 5 years after surgery, and they found that rates were not significantly different between patients with AL and patients without AL. Overall, there was a lack of comparing types of (re-)interventions.

## DISCUSSION

This systematic review appraised and synthesized the evidence on the impact of AL on QoL after oncological colorectal resections. In total, the studies comprised 4618 individual patients, with an overall incidence of AL of 12.4% (N = 572). QoL was assessed using 10 validated questionnaires administered at postoperative time points ranging from 1 month to 14 years. Overall, AL was found to negatively impact QoL at 6 and even 12 months postoperatively, with variable degrees of subsequent improvement.

The heterogeneity in questionnaires administered and variable times of assessment hindered our data analysis and may account for some conflicting results across studies. In a comprehensive systematic review of research studies on QoL and HRQoL, Haraldstad et al^33^ concluded that the majority experienced conceptual and methodological challenges with no clear consensus on how QoL should be measured. The use of various assessment tools and questionnaires in different studies hinders meaningful comparisons between similar study populations.^34^ Adoption of the standard set of outcomes for CRC proposed by the International Consortium for Health Outcomes Measurements (ICHOM) may avoid some of these issues.^35^ In this consortium, it was recommended to use the EORTC QLQ-C30 tool to capture overall QoL and the -CR29 to capture CRC-specific outcomes. The optimal time for QoL assessment was also addressed, with recommendations to administer questionnaires at baseline (before surgery), 6 months after surgery, and then annually for up to 10 years. Our research team suggests following the ICHOM recommendations.

Other patient and treatment variables such as the ASA score, BMI, anastomotic height, and adjuvant radiotherapy may impact QoL after CRC resections.^36–39^ Only 2 of the 13 included studies performed multivariate logistic regression analyses to investigate whether differences in QoL scores observed between AL and non-AL groups were due to the leak or driven by other factors such as neoadjuvant treatment, surgical procedure, or reintervention.^19,22^ Ideally, all studies should have performed such an analysis to verify whether AL is an independent factor that influences QoL. Besides, not all studies compare outcomes relative to baseline function, which weakens the interpretation of the functional scores at subsequent postoperative time points. As a result, it was difficult to draw valid conclusions comparing the included studies.

The observed decline in QoL scores reported among patients with AL in the first 6 and even 12 months may be due to several reasons. AL delays recovery and results in additional postoperative complications, higher rates of reintervention, and increased mortality within the first 30 days after surgery.^4,40^ This often prolongs the length of hospital stay and adversely impacts mobility and the ability of patients to care for themselves.^41–43^ Furthermore, some patients require stoma construction, which impairs role and social functioning scores.^44^ In the current study, there was a lack of correlation between stoma status and QoL outcomes. One study excluded patients with a stoma,^28^ whereas other studies did include them but did not draw strong conclusions on any association between stoma formation and QoL scores. AL has also been associated with higher rates of local recurrence and distant metastases in patients with CRC.^45,46^ Although smaller cohort studies have not found the same association between AL and colon cancer outcomes, the fear of (local) recurrence, as well as additional treatments required to mitigate the higher risk of recurrence, may further negatively impact QoL.^47,48^ Moreover, AL has been shown to be an independent risk factor for worse defecatory function (low anterior resection syndrome) and sexual function after CRC resections.^49–52^ Although these functional outcomes were not specifically assessed in the current study, it is crucial to consider their impact on overall QoL.^53^

To our knowledge, this is the first systematic review of the effects of AL on QoL in patients undergoing oncological colon and rectal resections. This study has limitations. First, a high heterogeneity in AL reporting was found in the included articles. It was often unclear what type of intervention and reoperation was performed to manage leaks. Because these elements are important when comparing outcomes, standardizing the reporting and management of leaks would be helpful. Subsequently, some studies only included patients with rectal cancer, whereas others included all types of colorectal surgeries. Second, a wide range of QoL questionnaires and time frames for assessment was used across the different studies. Although only studies using validated instruments were included, the heterogeneity of questionnaires created challenges when comparing outcomes across studies and interpreting results. The use of patient-centered methods, such as patient-reported outcome measures, may be even more informative to gain more insight into overall changes.^54^ Due to the heterogeneity of the included studies, comparisons across studies are limited and a meta-analysis was not possible to perform. Finally, all included studies demonstrated a moderate to serious risk of bias, which results in a low level of evidence, and caution is warranted by the presented findings.

## CONCLUSIONS

This systematic review demonstrated that QoL of patients with CRC may be compromised after AL up to 1 year, but assessment and reporting of QoL needs to be standardized to draw clear conclusions. In addition to exploring strategies for preventing and effectively managing AL, it is crucial to investigate long-term sequelae on patients’ QoL in future research. We recommend incorporating a standardized QoL assessment for patients with CRC who have experienced AL and integrating this outcome measure into a core outcome set for research focused on AL in the colorectal field. Continuous assessment and monitoring of QoL in patients undergoing CRC resection is essential to better support patients throughout their recovery. We emphasize the relevance of uniform reporting of AL outcomes to facilitate comparisons of results in future research. To reach this goal, we advise following the proposed questionnaires and time point as described by the CRC ICHOM working group,^35^ and work on a standardized reporting framework for AL-related research within the Consensus on Reporting and Defining Colorectal Anastomotic Leaks project.

## ACKNOWLEDGMENTS

The authors express their gratitude to Gregor Franssen, who ensured the appropriate search strategy as a professional clinical librarian at Maastricht University. They also thank the CoReAL Collaborative that has worked to highlight the QoL knowledge gap.

CoReAL Collaborative: Michel Adamina, Alberto Arezzo Mahdi Al-Taher, Tan Arulampalam, Saba Balvardi, Himani Bhatt, Marta Botti, Marylise Boutros, David A. Clark, Freek Daams, Jennifer S. Davids, Anse De Sadeleer, Abe Fingerhut, Nader Francis, Zoe Garoufalia, Roel Hompes, Neil H. Hyman, Mehraneh D. Jafari, John T. Jenkins, Audrey C.H.M. Jongen, Deborah S. Keller, Samuel H. Lai, Jérémie H. Lefevre, Bibi Martens, Justin A. Maykel, Jeongyoon Moon, Nariaki Okomoto, Ian Paquette, Gianluca Pellino, Sherief F. Shawki, Benjamin D. Shogan, Chelliah Selvasekar, Simon N.G. Siu Man, Jasper Stijns, Patricia Tejedor, William Tzu-Liang Chen, Christiaan van Der Leij, Steven D. Wexner, Elizabeth Wick, and Marina Yiasemidou.

## Supplementary Material



## References

[1] Nikolian VC Kamdar NS Regenbogen SE. Anastomotic leak after colorectal resection: a population-based study of risk factors and hospital variation. Surgery. 2017;161:1619–1627. 28238345 10.1016/j.surg.2016.12.033 PMC5433895

[2] 2015 European Society of Coloproctology collaborating group. The relationship between method of anastomosis and anastomotic failure after right hemicolectomy and ileo‐caecal resection: an international snapshot audit. Colorectal Dis. 2017;19:e296–e311. 10.1111/codi.13646 28263043

[3] Bakker I Grossmann I Henneman D Havenga K Wiggers T. Risk factors for anastomotic leakage and leak-related mortality after colonic cancer surgery in a nationwide audit. Br J Surg. 2014;101:424–432. 24536013 10.1002/bjs.9395

[4] Gessler B Eriksson O Angenete E. Diagnosis, treatment, and consequences of anastomotic leakage in colorectal surgery. Int J Colorectal Dis. 2017;32:549–556. 28070659 10.1007/s00384-016-2744-x PMC5355508

[5] Chadi SA Fingerhut A Berho M. Emerging trends in the etiology, prevention, and treatment of gastrointestinal anastomotic leakage. J Gastrointest Surg. 2016;20:2035–2051. 27638764 10.1007/s11605-016-3255-3

[6] Rahbari NN Weitz J Hohenberger W. Definition and grading of anastomotic leakage following anterior resection of the rectum: a proposal by the International Study Group of Rectal Cancer. Surgery. 2010;147:339–351. 20004450 10.1016/j.surg.2009.10.012

[7] Van Helsdingen CP Jongen AC De Jonge WJ Bouvy ND Derikx JP. Consensus on the definition of colorectal anastomotic leakage: a modified Delphi study. World J Gastroenterol. 2020;26:3293–3303. 32684743 10.3748/wjg.v26.i23.3293 PMC7336323

[8] Bruce J Krukowski ZH Al‐Khairy G Russell EM Park K. Systematic review of the definition and measurement of anastomotic leak after gastrointestinal surgery. Br J Surg. 2001;88:1157–1168. 11531861 10.1046/j.0007-1323.2001.01829.x

[9] Meyer J Naiken S Christou N. Reducing anastomotic leak in colorectal surgery: the old dogmas and the new challenges. World J Gastroenterol. 2019;25:5017–5025. 31558854 10.3748/wjg.v25.i34.5017 PMC6747296

[10] McDermott F Heeney A Kelly M Steele R Carlson G Winter D. Systematic review of preoperative, intraoperative and postoperative risk factors for colorectal anastomotic leaks. Br J Surg. 2015;102:462–479. 25703524 10.1002/bjs.9697

[11] Jongen AC Bosmans JW Kartal S. Predictive factors for anastomotic leakage after colorectal surgery: study protocol for a prospective observational study (REVEAL Study). JMIR Res Protoc. 2016;5:e90. 27282451 10.2196/resprot.5477 PMC4919551

[12] Bertelsen CA Andreasen A Jørgensen T Harling H Group DCC. Anastomotic leakage after curative anterior resection for rectal cancer: short and long‐term outcome. Colorectal Dis. 2010;12:e76–e81. 19438879 10.1111/j.1463-1318.2009.01935.x

[13] Page MJ McKenzie JE Bossuyt PM. The PRISMA 2020 statement: an updated guideline for reporting systematic reviews. Int J Surg. 2021;88:105906. 33789826 10.1016/j.ijsu.2021.105906

[14] The WHOQOL Group. The World Health Organization quality of life assessment (WHOQOL): development and general psychometric properties. Soc Sci Med. 1998;46:1569–1585. 9672396 10.1016/s0277-9536(98)00009-4

[15] Testa MA Simonson DC. Assessment of quality-of-life outcomes. N Engl J Med. 1996;334:835–840. 8596551 10.1056/NEJM199603283341306

[16] Snijders HS Wouters MW van Leersum NJ. Meta-analysis of the risk for anastomotic leakage, the postoperative mortality caused by leakage in relation to the overall postoperative mortality. Eur J Surg Oncol. 2012;38:1013–1019. 22954525 10.1016/j.ejso.2012.07.111

[17] Ouzzani M Hammady H Fedorowicz Z Elmagarmid A. Rayyan—a web and mobile app for systematic reviews. Syst Rev. 2016;5:1–10. 27919275 10.1186/s13643-016-0384-4 PMC5139140

[18] Sterne JA Hernán MA Reeves BC. ROBINS-I: a tool for assessing risk of bias in non-randomised studies of interventions. BMJ. 2016;355:i4919. 27733354 10.1136/bmj.i4919 PMC5062054

[19] Arron MN Custers JA van Goor H. The association between anastomotic leakage and health‐related quality of life after colorectal cancer surgery. Colorectal Dis. 2023;25:1381–1391. 36999516 10.1111/codi.16543

[20] Ashburn JH Stocchi L Kiran RP Dietz DW Remzi FH. Consequences of anastomotic leak after restorative proctectomy for cancer: effect on long-term function and quality of life. Dis Colon Rectum. 2013;56:275–280. 23392139 10.1097/DCR.0b013e318277e8a5

[21] Di Cristofaro L Ruffolo C Pinto E. Complications after surgery for colorectal cancer affect quality of life and surgeon–patient relationship. Colorectal Dis. 2014;16:O407–O419. 25155523 10.1111/codi.12752

[22] Hain E Manceau G Maggiori L Mongin C Prost À la Denise J Panis Y. Bowel dysfunction after anastomotic leakage in laparoscopic sphincter-saving operative intervention for rectal cancer: a case-matched study in 46 patients using the low anterior resection score. Surgery. 2017;161:1028–1039. 27894710 10.1016/j.surg.2016.09.037

[23] van Kooten RT van den Akker-Marle ME Putter H. The impact of postoperative complications on short-and long-term health-related quality of life after total mesorectal excision for rectal cancer. Clin Colorectal Cancer. 2022;21:325–338. 36210321 10.1016/j.clcc.2022.07.004

[24] Lim M Akhtar S Sasapu K. Clinical and subclinical leaks after low colorectal anastomosis: a clinical and radiologic study. Dis Colon Rectum. 2006;49:1611–1619. 16990979 10.1007/s10350-006-0663-6

[25] Marinatou A Theodoropoulos GE Karanika S. Do anastomotic leaks impair postoperative health-related quality of life after rectal cancer surgery? A case-matched study. Dis Colon Rectum. 2014;57:158–166. 24401876 10.1097/DCR.0000000000000040

[26] McGiffin T Clark DA Edmundson A Steffens D Stevenson A Solomon M. Surgical management and long‐term functional outcomes after anastomotic leak in patients undergoing minimally invasive restorative rectal resection and without a diverting ileostomy. ANZ J Surg. 2022;92:806–812. 35072326 10.1111/ans.17475

[27] Miura T Sakamoto Y Morohashi H Yoshida T Sato K Hakamada K. Risk factor for permanent stoma and incontinence quality of life after sphincter‐preserving surgery for low rectal cancer without a diverting stoma. Ann Gastroenterol Surg. 2018;2:79–86. 29863122 10.1002/ags3.12033 PMC5868869

[28] Mongin C Maggiori L Agostini J Ferron M Panis Y. Does anastomotic leakage impair functional results and quality of life after laparoscopic sphincter-saving total mesorectal excision for rectal cancer? A case-matched study. Int J Colorectal Dis. 2014;29:459–467. 24477790 10.1007/s00384-014-1833-y

[29] Di Re A Tooza S Diab J. Outcomes following anastomotic leak from rectal resections, including bowel function and quality of life. Ann Coloproctol. 2023;39:395–401. 35417955 10.3393/ac.2022.00073.0010 PMC10626330

[30] Riss S Stremitzer S Riss K Mittlböck M Bergmann M Stift A. Pelvic organ function and quality of life after anastomotic leakage following rectal cancer surgery. Wien Klin Wochen. 2011;123:53–57. 10.1007/s00508-010-1514-y 21191658

[31] Westerduin E Elfeki H Frontali A. Functional outcomes and quality of life after redo anastomosis in patients with rectal cancer: an international multicenter comparative cohort study. Dis Colon Rectum. 2021;64:822–832. 33902088 10.1097/DCR.0000000000002025

[32] Plastiras A Korkolis D Frountzas M Theodoropoulos G. The effect of anastomotic leak on postoperative pelvic function and quality of life in rectal cancer patients. Discov Oncol. 2022;13:52. 35751713 10.1007/s12672-022-00518-w PMC9233722

[33] Haraldstad K Wahl A Andenæs R. LIVSFORSK network. A systematic review of quality of life research in medicine and health sciences. *Qual Life Res*. 2019;28:2641–2650. 31187410 10.1007/s11136-019-02214-9 PMC6761255

[34] Freire Pequeno NP de Araújo Cabral NL Marchioni DM Vieira Cunha Lima SC de Oliveira Lyra C. Quality of life assessment instruments for adults: a systematic review of population-based studies. Health Qual Life Outcomes. 2020;18:208. 32605649 10.1186/s12955-020-01347-7 PMC7329518

[35] Zerillo JA Schouwenburg MG van Bommel ACM; Colorectal Cancer Working Group of the International Consortium for Health Outcomes Measurement (ICHOM). An international collaborative standardizing a comprehensive patient-centered outcomes measurement set for colorectal cancer. JAMA Oncol. 2017;3:686–694. 28384684 10.1001/jamaoncol.2017.0417

[36] Birgisson H Påhlman L Gunnarsson U Glimelius B; Swedish Rectal Cancer Trial Group. Adverse effects of preoperative radiation therapy for rectal cancer: long-term follow-up of the Swedish Rectal Cancer Trial. J Clin Oncol. 2005;23:8697–8705. 16314629 10.1200/JCO.2005.02.9017

[37] Cummings A Grimmett C Calman L; Members of CREW Study Advisory Committee. Comorbidities are associated with poorer quality of life and functioning and worse symptoms in the 5 years following colorectal cancer surgery: results from the ColoREctal Well-being (CREW) cohort study. Psychooncology. 2018;27:2427–2435. 30070052 10.1002/pon.4845 PMC6221152

[38] Schlesinger S Walter J Hampe J. Lifestyle factors and health-related quality of life in colorectal cancer survivors. Cancer Causes Control. 2014;25:99–110. 24158780 10.1007/s10552-013-0313-y

[39] Tsunoda A Nakao K Tsunoda Y Watanabe M Matsui N. Health-related quality of life of colorectal cancer patients receiving oral UFT plus leucovorin compared with those with surgery alone. Int J Clin Oncol. 2010;15:153–160. 20191299 10.1007/s10147-010-0035-z

[40] Sciuto A Merola G De Palma GD. Predictive factors for anastomotic leakage after laparoscopic colorectal surgery. World J Gastroenterol. 2018;24:2247–2260. 29881234 10.3748/wjg.v24.i21.2247 PMC5989239

[41] Borghi F Migliore M Cianflocca D; Italian ColoRectal Anastomotic Leakage (iCral) study group. Management and 1-year outcomes of anastomotic leakage after elective colorectal surgery. Int J Colorectal Dis. 2021;36:929–939. 33118101 10.1007/s00384-020-03777-7

[42] Hammond J Lim S Wan Y Gao X Patkar A. The burden of gastrointestinal anastomotic leaks: an evaluation of clinical and economic outcomes. J Gastrointest Surg. 2014;18:1176–1185. 24671472 10.1007/s11605-014-2506-4 PMC4028541

[43] Brown SR Mathew R Keding A Marshall HC Brown JM Jayne DG. The impact of postoperative complications on long-term quality of life after curative colorectal cancer surgery. Ann Surg. 2014;259:916–923. 24374539 10.1097/SLA.0000000000000407

[44] Herrle F Sandra-Petrescu F Weiss C Post S Runkel N Kienle P. Quality of life and timing of stoma closure in patients with rectal cancer undergoing low anterior resection with diverting stoma: a multicenter longitudinal observational study. Dis Colon Rectum. 2016;59:281–290. 26953986 10.1097/DCR.0000000000000545

[45] Krarup P-M Nordholm-Carstensen A Jorgensen LN Harling H. Anastomotic leak increases distant recurrence and long-term mortality after curative resection for colonic cancer: a nationwide cohort study. Ann Surg. 2014;259:930–938. 24045445 10.1097/SLA.0b013e3182a6f2fc

[46] Ha GW Kim JH Lee MR. Oncologic impact of anastomotic leakage following colorectal cancer surgery: a systematic review and meta-analysis. Ann Surg Oncol. 2017;24:3289–3299. 28608118 10.1245/s10434-017-5881-8

[47] Lim CYS Laidsaar‐Powell RC Young JM Kao SCH Zhang Y Butow P. Colorectal cancer survivorship: a systematic review and thematic synthesis of qualitative research. Eur J Cancer Care (Engl). 2021;30:e13421. 33733545 10.1111/ecc.13421

[48] Lim CYS Laidsaar-Powell RC Young JM; advanced-CRC survivorship authorship group. Fear of cancer progression and death anxiety in survivors of advanced colorectal cancer: a qualitative study exploring coping strategies and quality of life. OMEGA. 2022;0:302228221121493. 10.1177/00302228221121493 36127158

[49] Khomyakov EA Nafedzov IO Fomenko OY. Risk factors for major low anterior resection syndrome: meta-analysis and systematic literature review. Russian Open Med J. 2021;10:113.

[50] Sun R Dai Z Zhang Y Lu J Zhang Y Xiao Y. The incidence and risk factors of low anterior resection syndrome (LARS) after sphincter-preserving surgery of rectal cancer: a systematic review and meta-analysis. Support Care Cancer. 2021;29:7249–7258. 34296335 10.1007/s00520-021-06326-2

[51] Hultberg DK Svensson J Jutesten H. The impact of anastomotic leakage on long-term function after anterior resection for rectal cancer. Dis Colon Rectum. 2020;63:619–628. 32032197 10.1097/DCR.0000000000001613

[52] Lange M Marijnen C Maas C. Dutch CCIot: risk factors for sexual dysfunction after rectal cancer treatment. Eur J Cancer. 2009;45:1578–1588. 19147343 10.1016/j.ejca.2008.12.014

[53] Vironen JH Kairaluoma M Aalto AM Kellokumpu IH. Impact of functional results on quality of life after rectal cancer surgery. Dis Colon Rectum. 2006;49:568–578. 16583289 10.1007/s10350-006-0513-6

[54] Cella D Riley W Stone A; PROMIS Cooperative Group. The Patient-Reported Outcomes Measurement Information System (PROMIS) developed and tested its first wave of adult self-reported health outcome item banks: 2005–2008. J Clin Epidemiol. 2010;63:1179–1194. 20685078 10.1016/j.jclinepi.2010.04.011 PMC2965562

