# Supplementary material for: Impact of Anastomotic Leakage After Colorectal Cancer Surgery on Quality of Life: A Systematic Review
Source: Dis Colon Rectum. 2024 Oct 23;68(2):154–70. doi: 10.1097/DCR.0000000000003478 (PMC11748909; doi:10.1097/DCR.0000000000003478)
Supplement: Supplementary file 1 [file dcr-68-154-s001.pdf]

Supplementary

S1. Search Strategy

Pubmed	((("Colorectal Neoplasms"[MeSH] OR (("Neoplasms"[MeSH] OR carcinoma*[tiab] OR adenocarcinoma*[tiab] OR neoplas*[tiab] OR tumour*[tiab] OR tumor*[tiab] OR oncolog*[tiab] OR malignan*[tiab] OR cancer*[tiab]) AND (colorectal*[tiab] OR colon[tiab] OR colonic[tiab] OR rectal[tiab] OR rectum[tiab] OR sigmoid*[tiab]))) AND ("Colectomy"[MeSH] OR "Colorectal Surgery"[MeSH] OR "Rectum/surgery"[MeSH] OR "Colon/surgery"[MeSH] OR ((large bowel[tiab] OR colorectal*[tiab] OR colon[tiab] OR rectum[tiab] OR rectal[tiab] OR ileocaecal[tiab] OR caecum[tiab] OR low anterior[tiab]) AND (resection*[tiab] OR surg*[tiab] OR anastomo*[tiab] OR "Anastomosis, Surgical"[MeSH])) OR (colectom*[tiab] OR hemicolectom*[tiab] OR "total mesorectal excision*"[tiab] OR proctocolectom*[tiab] OR "abdominal perineal resection*"[tiab]))) AND ("Anastomotic Leak"[MeSH] OR (anastomo*[tiab] AND ("adverse effects"[Subheading] OR "complications"[Subheading] OR leak*[tiab] OR complication*[tiab] OR defect*[tiab] OR separation*[tiab] OR dehiscence*[tiab] OR breakdown*[tiab] OR abscess*[tiab])))) AND ("Quality of Life"[Mesh] OR "quality of life"[tiab] OR life qualit*[tiab] OR living qualit*[tiab] OR "quality of living"[tiab] OR "Activities of Daily Living"[Mesh] OR "activities of daily living"[tiab] OR "activity of daily living"[tiab] OR "activities of daily life"[tiab] OR "activity of daily life"[tiab] OR daily living activit*[tiab] OR daily life activit*[tiab] OR "adl"[tiab] OR "chronic limitation of activity"[tiab] OR self care*[tiab] OR "Health Status"[Mesh] OR "health status"[tiab] OR "level of health"[tiab] OR health level*[tiab] OR "qol"[tiab] OR "hrql"[tiab] OR "hrqol"[tiab] OR "activity of daily living"[tiab] OR "activities of daily life"[tiab] OR "activity of daily life"[tiab] OR daily life activit*[tiab] OR iadl[tiab] OR living qualit*[tiab] OR "quality of living"[tiab] OR "patient reported outcome")
Cochrane	("Colorectal Neoplasms"[MeSH] OR (("Neoplasms"[MeSH] OR carcinoma*:ti,ab,kw OR adenocarcinoma*:ti,ab,kw OR neoplas*:ti,ab,kw OR tumour*:ti,ab,kw OR tumor*:ti,ab,kw OR oncolog*:ti,ab,kw OR malignan*:ti,ab,kw OR cancer*:ti,ab,kw) AND (colorectal*:ti,ab,kw OR colon:ti,ab,kw OR colonic:ti,ab,kw OR rectal:ti,ab,kw OR rectum:ti,ab,kw OR sigmoid*:ti,ab,kw))) AND ("Colectomy"[MeSH] OR "Colorectal Surgery"[MeSH] OR "Rectum/surgery"[MeSH] OR "Colon/surgery"[MeSH] OR ((large bowel:ti,ab,kw OR colorectal*:ti,ab,kw OR colon:ti,ab,kw OR rectum:ti,ab,kw OR rectal:ti,ab,kw OR ileocaecal:ti,ab,kw OR caecum:ti,ab,kw OR low anterior:ti,ab,kw) AND (resection*:ti,ab,kw OR surg*:ti,ab,kw OR anastomo*:ti,ab,kw OR "Anastomosis, Surgical"[MeSH])) OR (colectom*:ti,ab,kw OR hemicolectom*:ti,ab,kw OR "total mesorectal excision*":ti,ab,kw OR proctocolectom*:ti,ab,kw OR "abdominal perineal resection*":ti,ab,kw)) AND ("Anastomotic Leak"[MeSH] OR (anastomo*:ti,ab,kw AND ("adverse effects"[Subheading] OR "complications"[Subheading] OR leak*:ti,ab,kw OR complication*:ti,ab,kw OR defect*:ti,ab,kw OR separation*:ti,ab,kw OR dehiscence*:ti,ab,kw OR breakdown*:ti,ab,kw OR abscess*:ti,ab,kw)) AND (“Quality of Life”[MeSH] OR (Quality of life OR QoL OR life quality OR health-related quality of life OR HRQoL OR Psychological Well-Being OR health level OR health status OR daily life OR patient reported outcome):ti,ab,kw)
Embase	(exp colorectal tumor/ or exp colorectal cancer/ or ((neoplasm/ or (carcinoma* or adenocarcinoma* or neoplas* or tumour* or tumor* or oncolog* or malignan* or cancer*).ti,ab,kw.) adj3 (colorectal* or colon or colonic or rectal or rectum or sigmoid*).ti,ab,kw.)) AND (exp colorectal surgery/ or exp rectum surgery/ or exp colon surgery/ or ileoanal anastomosis/ or ileorectal anastomosis/ or ((large bowel or colorectal* or colon or rectum or rectal or ileocaecal or caecum or low anterior).ti,ab,kw. adj3 (resection* or surg* or anastomo*).ti,ab,kw. or (colectom* or hemicolectom* or "total mesorectal excision*" or proctocolectom* or "abdominal perineal resection*").ti,ab,kw.)) AND (postoperative complication/su or exp anastomosis leakage/ or anastomosis/co or (anastomo* adj3 (leak* or complication*)).ti,ab,kw.) AND (exp "quality of life"/ OR "life qualit*".ti,ab,kw. OR "quality o? life*".ti,ab,kw. OR exp health status/ OR "health status*".ti,ab,kw. OR "level o? health".ti,ab,kw. OR "health level*".ti,ab,kw. OR qol.ti,ab,kw. OR hrql.ti,ab,kw. OR hrqol.ti,ab,kw. OR "patient reported outcome".ti,ab,kw.)

S2. Additional study information on perioperative care

Reference	Bowel preparation (pre-op)	Oral antibiotics (pre-op)	Diverting ileostomy (intra-op)	Drain management (intra-op)
**Arron et al.**	NA	NA	If needed	NA
**Ashburn et al.**	NA	NA	Based on the discretion of the operating surgeon	NA
**di Cristofaro et al.**	NA	NA	NA	NA
**Hain et al.**	NA	NA	Performed routinely and closed 6 to 8 weeks postoperatively if CT showed no AL	Drainage of the pelvic space by a closed suction drain was left in place for 48–72 h in all patients
**van Kooten et al.**	NA	NA	NA	NA
**Lim et al**	NA	prophylaxis of 1.5 g of cefuroxime and 500 mg of metronidazole at induction	Based on the discretion of the senior surgeon	NA
**Marinatou et al.**	NA	NA	Preferred by the surgeon in the majority of the cases where a low-lying anastomosis was necessary	Not clear if this was done routinely or in selected cases
**McGiffin et al.**	NA	NA	Not performed, exclusion criterium	NA
**Miura et al.**	Mechanical bowel preparation	Perioperative	Not performed, exclusion criterium	Transanal drainage
**Mongin et al.**	NA	NA	Performed routinely and closed 6 to 8 weeks postoperatively if CT showed no AL	A pelvic suction drain was always left in place behind the anastomosis
**di Re et al.**	NA	NA	Based on the surgeon's discretion	NA
**Riss et al.**	NA	NA	NA	NA
**Westerduin et al.**	NA	NA	If deemed necessary by the operating surgeon	NA

S3. Characteristics of anastomotic leakage described by the included studies

Reference	Definition AL	Timeframe for AL diagnosis	Clinical characteristics	Radiological characteristics	Indication for radiological examination/AL assessment	Biochemical elements	Grading system	Re-interventions	Mortality *<30 post-operative days*
Arron et al.	AL was defined as requiring re-intervention within 30 days following surgery	30 days	NA	NA	Based on clinical symptoms	NA	ISREC classification Grade B & C	- Radiological intervention (n=4)<br>- Re-operation (n=56)<br>- Other (i.e. conservative) (n=3)	NA
Ashburn et al.	Clinical, radiographic, or intraoperative findings guided the primary surgeon to document AL in the medical record and only patients with clinically evident AL were included.	NA	Presence of pus, gas or stool evacuated in a drain, similar discharge from the operative wound, or a rectovaginal fistula or peritonitis.	Distal contrast study	Only patients with clinical symptoms were included	NA	NA	NA	NA
di Cristofaro et al.	NA	NA	NA	NA	NA	NA	Clavien Dindo, Grade III	Reoperation (n = 5)	0
Hain et al.	Symptomatic AL or asymptomatic AL	> 6 months	Signs of sepsis, peritonitis, emission of gas, pus or faeces from the pelvic drain, purulent discharge per anus, or rectovaginal fistula.	CT with water-soluble contrast enema: luminal extravasation or isolated pelvic abscess	Based on clinical symptoms or routine performed at 6–8 weeks postoperatively for ileostomy closure	NA	Symptomatic (n=23) or asymptomatic AL (n=23)	- Delayed stoma reversal (all)<br>- Antibiotic therapy (n=23)<br>- Radiological drainage (n=9)<br>- Trans anal drainage (n=3)<br>- Trans anal dilatation (n=3)<br>- Abdominal re-intervention (n=3)<br>- Fibrin glue injection (n=1)	NA

**Reference**	**Definition AL**	**Timeframe for AL diagnosis**	**Clinical characteristics**	**Radiological characteristics**	**Indication for radiological examination/AL assessment**	**Biochemical elements**	**Grading system**	**Re-interventions**	**Mortality** *<30 post-operative days*
van Kooten et al.	All leakages clinically diagnosed or confirmed by contrast enema, or an abscess around the anastomosis	NA	NA	Contrast enema, abscess around the anastomosis	NA	NA	NA	NA	Mortality within 30 days was an exclusion criterion
Lim et al.	Clinical or subclinical AL	< 3 months	Abdominal pain, distention or tenderness, peritonism, rectal discharge, tachycardia, arrhythmias or pyrexia.	'Confirmation' with CT scanning and/or stricture or leak on water-soluble contrast enema	Based on clinical symptoms or routine performed within three months for ileostomy closure	Elevated white cell count or elevated serum C-reactive protein	Clinical vs subclinical AL	- Laparotomy and washout (n=1)<br>- CT-guided drainage (n=3)<br>- Antibiotic therapy (n=6)	NA
Marinatou et al.	All clinically evident ALs	NA	Peritonitis, purulent and/or feculent material from pelvic drain, fever with or without pelvic pain, protracted ileus, drainage per rectum and rectovaginal fistula	Confirmation of the anastomotic insufficiency obtained by CT and/or contrast medium exploration (gastrografin)	Based on clinical symptoms	NA	Clavien Dindo<br>- Grade II: 14<br>- Grade III: 11	- CT-guided placement of presacral drain (n=1)<br>- Re-laparotomy with pelvic drain placement (n=2)<br>- Re-laparotomy with loop ileostomy (n=3)<br>- Re-laparotomy with takedown of anastomosis and end colostomy construction (n=5)	0
McGiffin et al.	ISREC definition*	Median 5.5 days POD	NA	NA	NA	NA	ISREC classification:<br>Grade A (n=0)<br>Grade B (n=2)<br>Grade C (n=22)	- Percutaneous drain (n=2)<br>- Laparoscopic washout, without ileostomy (n=1)<br>- Laparoscopic washout, with ileostomy (n=17)<br>- Open washout, with ileostomy (n=4)	0

Reference	Definition AL	Timeframe for AL diagnosis	Clinical characteristics	Radiological characteristics	Indication for radiological examination/AL assessment	Biochemical elements	Grading system	Re-interventions	Mortality *<30 post-operative days*
Miura et al.	ISREC definition	< 30 days	Abdominal pain, abdominal distention, fever, pus or faecal discharge from the pelvic drain. Confirmed by digital rectal examination	Extravasation of endoluminally given water-soluble contrast enema, pelvic abscess and fluid/air bubbles surrounding the anastomosis on CT	Based on clinical symptoms	NA	ISREC classification	- Re-intervention: 30<br>- Reoperation: 30	One patient died because of AL
Mongin et al.	NA	NA	Peritonitis, fever, discharge of gas, pus or faeces from the abdominal drain or the vagina	CT scan with contrast enema with leakage into the perirectal cavity, leakages from the efferent limb of the colonic pouch, isolated pelvic abscesses without evidence of fistula	Based on clinical symptoms or routine performed at 6–8 weeks postoperatively for ileostomy closure	NA	Symptomatic AL (n=11) vs asymptomatic AL (n=41).	- Antibiotic therapy (n=11)<br>- CT-guided drainage (n=9)<br>- Trans anal drainage (n=2)<br>- Reoperation (n=13)	0
di Re et al.	Leak in the bowel anastomosis that is identified at reoperation, post-operative imaging or evidence of anastomotic stenosis on routine endoscopic examination, clinical or radiological examination	NA	Stenosis	Presence of extraluminal gas or peri-anastomotic collection on post-operative imaging	NA	NA	Contained (66%) vs not contained (10%) and stricture only (24%)	- Antibiotics only (39%)<br>- Percutaneous radiological drainage (9%)<br>- Operative abdominal drainage (19%)<br>- Transrectal drainage (6%)<br>- Anastomotic dilatation (24%)<br>- Combined percutaneous and transrectal drainage (2%)<br>- Combination of abdominal and	0

Reference	Definition AL	Timeframe for AL diagnosis	Clinical characteristics	Radiological characteristics	Indication for radiological examination/AL assessment	Biochemical elements	Grading system	Re-interventions	Mortality *<30 post-operative days*
								transrectal drainage (1%)	
Riss et al.	NA	NA	NA	NA	NA	NA	ISREC classification: Grade A (n=7) Grade B (n=3) Grade C (n=6)	Re-laparotomy in 6 patients.	NA
Westerduin et al.	AL, pelvic abscess or fistula	NA	NA	NA	NA	NA	NA	Re-do anastomosis (surgery)	0

AL, anastomotic leakage; NA, not applicable; CT, Computed Tomography; ISREC, the International Study Group of Rectal Cancer; POD, postoperative days.
**'A defect of the intestinal wall integrity at the colorectal or colo-anal anastomotic site leading to a communication between the intra- and extra-luminal compartments'*

S4. Detailed information of questionnaires used in more than one study

a. EORTC QLQ-CR29 – downloaded from https://qol.eortc.org/questionnaire/qlq-cr29/

ENGLISH

EORTC QLQ – CR29

Patients sometimes report that they have the following symptoms or problems. Please indicate the extent to which you have experienced these symptoms or problems during the past week. Please answer by circling the number that best applies to you.

During the past week:	Not at All	A Little	Quite a Bit	Very Much
31. Did you urinate frequently during the day?	1	2	3	4
32. Did you urinate frequently during the night?	1	2	3	4
33. Have you had any unintentional release (leakage) of urine?	1	2	3	4
34. Did you have pain when you urinated?	1	2	3	4
35. Did you have abdominal pain?	1	2	3	4
36. Did you have pain in your buttocks/anal area/rectum?	1	2	3	4
37. Did you have a bloated feeling in your abdomen?	1	2	3	4
38. Have you had blood in your stools?	1	2	3	4
39. Have you had mucus in your stools?	1	2	3	4
40. Did you have a dry mouth?	1	2	3	4
41. Have you lost hair as a result of your treatment?	1	2	3	4
42. Have you had problems with your sense of taste?	1	2	3	4

During the past week:	Not at All	A Little	Quite a Bit	Very Much
43. Were you worried about your health in the future?	1	2	3	4
44. Have you worried about your weight?	1	2	3	4
45. Have you felt physically less attractive as a result of your disease or treatment?	1	2	3	4
46. Have you been feeling less feminine/masculine as a result of your disease or treatment?	1	2	3	4
47. Have you been dissatisfied with your body?	1	2	3	4
48. Do you have a stoma bag (colostomy/ileostomy)? (please circle the correct answer)	Yes		No	

Please go on to the next page

ENGLISH

During the past week:	Not at All	A Little	Quite a Bit	Very Much
**Answer these questions ONLY IF YOU HAVE A STOMA BAG, if not please continue below:**				
49. Have you had unintentional release of gas/flatulence from your stoma bag?	1	2	3	4
50. Have you had leakage of stools from your stoma bag?	1	2	3	4
51. Have you had sore skin around your stoma?	1	2	3	4
52. Did frequent bag changes occur during the day?	1	2	3	4
53. Did frequent bag changes occur during the night?	1	2	3	4
54. Did you feel embarrassed because of your stoma?	1	2	3	4
55. Did you have problems caring for your stoma?	1	2	3	4
**Answer these questions ONLY IF YOU DO NOT HAVE A STOMA BAG:**				
49. Have you had unintentional release of gas/flatulence from your back passage?	1	2	3	4
50. Have you had leakage of stools from your back passage?	1	2	3	4
51. Have you had sore skin around your anal area?	1	2	3	4
52. Did frequent bowel movements occur during the day?	1	2	3	4
53. Did frequent bowel movements occur during the night?	1	2	3	4
54. Did you feel embarrassed because of your bowel movement?	1	2	3	4

During the past 4 weeks:	Not at All	A Little	Quite a Bit	Very Much
**For men only:**				
56. To what extent were you interested in sex?	1	2	3	4
57. Did you have difficulty getting or maintaining an erection?	1	2	3	4
**For women only:**				
58. To what extent were you interested in sex?	1	2	3	4
59. Did you have pain or discomfort during intercourse?	1	2	3	4

© QLQ-CR29 Copyright 2006 EORTC Quality of Life Group. All rights reserved. Version 2.1

SPECIMEN

SPECIMEN

b. EORTC QLQ-C30 – downloaded from https://qol.eortc.org/questionnaire/eortc-qlq-c30/

ENGLISH

EORTC QLQ-C30 (version 3)

We are interested in some things about you and your health. Please answer all of the questions yourself by circling the number that best applies to you. There are no "right" or "wrong" answers. The information that you provide will remain strictly confidential.

Please fill in your initials: |_|_|_|_|

Your birthdate (Day, Month, Year): |_|_|_|_|_|_|_|_|

Today's date (Day, Month, Year): 31 |_|_|_|_|_|_|_|_|

		Not at All	A Little	Quite a Bit	Very Much
1.	Do you have any trouble doing strenuous activities, like carrying a heavy shopping bag or a suitcase?	1	2	3	4
2.	Do you have any trouble taking a long walk?	1	2	3	4
3.	Do you have any trouble taking a short walk outside of the house?	1	2	3	4
4.	Do you need to stay in bed or a chair during the day?	1	2	3	4
5.	Do you need help with eating, dressing, washing yourself or using the toilet?	1	2	3	4

During the past week:

		Not at All	A Little	Quite a Bit	Very Much
6.	Were you limited in doing either your work or other daily activities?	1	2	3	4
7.	Were you limited in pursuing your hobbies or other leisure time activities?	1	2	3	4
8.	Were you short of breath?	1	2	3	4
9.	Have you had pain?	1	2	3	4
10.	Did you need to rest?	1	2	3	4
11.	Have you had trouble sleeping?	1	2	3	4
12.	Have you felt weak?	1	2	3	4
13.	Have you lacked appetite?	1	2	3	4
14.	Have you felt nauseated?	1	2	3	4
15.	Have you vomited?	1	2	3	4
16.	Have you been constipated?	1	2	3	4

Please go on to the next page

SPECIMEN

ENGLISH

During the past week:

		Not at All	A Little	Quite a Bit	Very Much
17.	Have you had diarrhea?	1	2	3	4
18.	Were you tired?	1	2	3	4
19.	Did pain interfere with your daily activities?	1	2	3	4
20.	Have you had difficulty in concentrating on things, like reading a newspaper or watching television?	1	2	3	4
21.	Did you feel tense?	1	2	3	4
22.	Did you worry?	1	2	3	4
23.	Did you feel irritable?	1	2	3	4
24.	Did you feel depressed?	1	2	3	4
25.	Have you had difficulty remembering things?	1	2	3	4
26.	Has your physical condition or medical treatment interfered with your family life?	1	2	3	4
27.	Has your physical condition or medical treatment interfered with your social activities?	1	2	3	4
28.	Has your physical condition or medical treatment caused you financial difficulties?	1	2	3	4

For the following questions please circle the number between 1 and 7 that best applies to you

29. How would you rate your overall health during the past week?

1	2	3	4	5	6	7
Very poor						Excellent

30. How would you rate your overall quality of life during the past week?

1	2	3	4	5	6	7
Very poor						Excellent

SPECIMEN

© Copyright 1995 EORTC Quality of Life Group. All rights reserved. Version 3.0

c. SF-36 – downloaded from https://www.rand.org/health-care/surveys_tools/mos/36-item-short-form.html

RAND HEALTH

RAND > RAND Health > Surveys > RAND Medical Outcomes Study > 36-Item Short Form Survey (SF-36) >

36-Item Short Form Survey Instrument (SF-36)

RAND 36-Item Health Survey 1.0 Questionnaire Items

Choose one option for each questionnaire item.

1. In general, would you say your health is:

- 1 - Excellent
- 2 - Very good
- 3 - Good
- 4 - Fair
- 5 - Poor

2. **Compared to one year ago**, how would you rate your health in general **now**?

- 1 - Much better now than one year ago
- 2 - Somewhat better now than one year ago
- 3 - About the same
- 4 - Somewhat worse now than one year ago
- 5 - Much worse now than one year ago

The following items are about activities you might do during a typical day. Does **your health now limit you** in these activities? If so, how much?

	Yes, limited a lot	Yes, limited a little	No, not limited at all
3. **Vigorous activities**, such as running, lifting heavy objects, participating in strenuous sports	1	2	3
4. **Moderate activities**, such as moving a table, pushing a vacuum cleaner, bowling, or playing golf	1	2	3
5. Lifting or carrying groceries	1	2	3
6. Climbing **several** flights of stairs	1	2	3
7. Climbing **one** flight of stairs	1	2	3
8. Bending, kneeling, or stooping	1	2	3
9. Walking **more than a mile**	1	2	3
10. Walking **several blocks**	1	2	3
11. Walking **one block**	1	2	3
12. Bathing or dressing yourself	1	2	3

During the **past 4 weeks**, have you had any of the following problems with your work or other regular daily activities **as a result of your physical health**?

	Yes	No
13. Cut down the **amount of time** you spent on work or other activities	1	2
14. **Accomplished less** than you would like	1	2
15. Were limited in the **kind** of work or other activities	1	2
16. Had **difficulty** performing the work or other activities (for example, it took extra effort)	1	2

During the **past 4 weeks**, have you had any of the following problems with your work or other regular daily activities **as a result of any emotional problems** (such as feeling depressed or anxious)?

	Yes	No
17. Cut down the **amount of time** you spent on work or other activities	1	2
18. **Accomplished less** than you would like	1	2
19. Didn't do work or other activities as **carefully** as usual	1	2

20. During the **past 4 weeks**, to what extent has your physical health or emotional problems interfered with your normal social activities with family, friends, neighbors, or groups?

- 1 - Not at all
- 2 - Slightly
- 3 - Moderately
- 4 - Quite a bit
- 5 - Extremely

21. How much **bodily** pain have you had during the **past 4 weeks**?

- 1 - None
- 2 - Very mild
- 3 - Mild
- 4 - Moderate
- 5 - Severe
- 6 - Very severe

22. During the **past 4 weeks**, how much did **pain** interfere with your normal work (including both work outside the home and housework)?

- 1 - Not at all
- 2 - A little bit
- 3 - Moderately
- 4 - Quite a bit
- 5 - Extremely

These questions are about how you feel and how things have been with you **during the past 4 weeks**. For each question, please give the one answer that comes closest to the way you have been feeling.

How much of the time during the **past 4 weeks**...

	All of the time	Most of the time	A good bit of the time	Some of the time	A little of the time	None of the time
23. Did you feel full of pep?	1	2	3	4	5	6
24. Have you been a very nervous person?	1	2	3	4	5	6
25. Have you felt so down in the dumps that nothing could cheer you up?	1	2	3	4	5	6
26. Have you felt calm and peaceful?	1	2	3	4	5	6
27. Did you have a lot of energy?	1	2	3	4	5	6
28. Have you felt downhearted and blue?	1	2	3	4	5	6
29. Did you feel worn out?	1	2	3	4	5	6
30. Have you been a happy person?	1	2	3	4	5	6
31. Did you feel tired?	1	2	3	4	5	6

32. During the **past 4 weeks**, how much of the time has **your physical health or emotional problems** interfered with your social activities (like visiting with friends, relatives, etc.)?

- 1 - All of the time
- 2 - Most of the time
- 3 - Some of the time
- 4 - A little of the time
- 5 - None of the time

How TRUE or FALSE is **each** of the following statements for you.

	Definitely true	Mostly true	Don't know	Mostly false	Definitely false
33. I seem to get sick a little easier than other people	1	2	3	4	5
34. I am as healthy as anybody I know	1	2	3	4	5
35. I expect my health to get worse	1	2	3	4	5
36. My health is excellent	1	2	3	4	5

ABOUT

The RAND Corporation is a research organization that develops solutions to public policy challenges to help make communities throughout the world safer and more secure, healthier and more prosperous. RAND is nonprofit, nonpartisan, and committed to the public interest.

1776 Main Street
Santa Monica, California 90401-3208

RAND® is a registered trademark. Copyright © 1994-2016 RAND Corporation.

d. FIQL – downloaded from https://patientreportedoutcomes2.sites.olt.ubc.ca/files/2014/04/Fecal_Incontinence_Quality_of_Life_Paper.pdf

Q 1: In general, would you say your health is:

1 ☐ Excellent
2 ☐ Very Good
3 ☐ Good
4 ☐ Fair
5 ☐ Poor

Q 2: For each of the items, please indicate how much of the time the issue is a concern for you due to accidental bowel leakage. (If it is a concern for you for reasons other than accidental bowel leakage then check the box under Not Apply, (N/A).)

Q2. Due to accidental bowel leakage:	Most of the Time	Some of The Time	A Little of the Time	None of the Time	N/A
a. I am afraid to go out	1	2	3	4	☐
b. I avoid visiting friends	1	2	3	4	☐
c. I avoid staying overnight away from home	1	2	3	4	☐
d. It is difficult for me to get out and do things like going to a movie or to church	1	2	3	4	☐
e. I cut down on how much I eat before I go out	1	2	3	4	☐
f. Whenever I am away from home, I try to stay near a restroom as much as possible	1	2	3	4	☐
g. It is important to plan my schedule (daily activities) around my bowel pattern	1	2	3	4	☐
h. I avoid traveling	1	2	3	4	☐
i. I worry about not being able to get to the toilet in time	1	2	3	4	☐
j. I feel I have no control over my bowels	1	2	3	4	☐
k. I can't hold my bowel movement long enough to get to the bathroom	1	2	3	4	☐
l. I leak stool without even knowing it	1	2	3	4	☐
m. I try to prevent bowel accidents by staying very near a bathroom	1	2	3	4	☐

Q 3: Due to accidental bowel leakage, indicate the extent to which you AGREE or DISAGREE with each of the following items. (If it is a concern for you for reasons other than accidental bowel leakage then check the box under Not Apply, N/A).

Q3. Due to accidental bowel leakage:	Strongly Agree	Somewhat Agree	Somewhat Disagree	Strongly Disagree	N/A
a. I feel ashamed	1	2	3	4	☐
b. I can not do many of things I want to do	1	2	3	4	☐
c. I worry about bowel accidents	1	2	3	4	☐
d. I feel depressed	1	2	3	4	☐
e. I worry about others smelling stool on me	1	2	3	4	☐
f. I feel like I am not a healthy person	1	2	3	4	☐
g. I enjoy life less	1	2	3	4	☐
h. I have sex less often than I would like to	1	2	3	4	☐
i. I feel different from other people	1	2	3	4	☐
j. The possibility of bowel accidents is always on my mind	1	2	3	4	☐
k. I am afraid to have sex	1	2	3	4	☐
l. I avoid traveling by plane or train	1	2	3	4	☐
m. I avoid going out to eat	1	2	3	4	☐
n. Whenever I go someplace new, I specifically locate where the bathrooms are	1	2	3	4	☐

Q 4: During the past month, have you felt so sad, discouraged, hopeless, or had so many problems that you wondered if anything was worthwhile?

1 ☐ Extremely So - To the point that I have just about given up
2 ☐ Very Much So
3 ☐ Quite a Bit
4 ☐ Some - Enough to bother me
5 ☐ A Little Bit
6 ☐ Not At All

Scale Scoring

Scales range from 1 to 5, with a 1 indicating a lower functional status of quality of life. Scale scores are the average (mean) response to all items in the scale (*e.g.*, add the responses to all questions in a scale together and then divide by the number of items in the scale. Not Apply is coded as a missing value in the analysis for all questions.)

Scale 1. Lifestyle, ten items: Q2a Q2b Q2c Q2d Q2e Q2g Q2h Q3b Q3l Q3m

Scale 2. Coping/Behavior, nine items: Q2f Q2i Q2j Q2k Q2m Q3d Q3h Q3j Q3n

Scale 3. Depression/Self Perception, seven items: Q1 Q3d Q3f Q3g Q3i Q3k Q4, (Question 1 is reverse coded.)

Scale 4. Embarrassment, three items: Q2l Q3a Q3e

S5. Links to questionnaires used in only one study

a. Cleveland Global QoL: https://cdn-links.lww.com/permalink/ibd/b/ibd_2015_07_09_shen_15-0060_sdc1.pdf
b. EORTC IN – PATSAT32: https://www.eortc.org/app/uploads/sites/2/2018/08/Specimen-IN-PATSAT32-English.pdf
c. Gastrointestinal Quality of Life Index: https://patientreportedoutcomes2.sites.olt.ubc.ca/files/2014/04/GQLI.pdf
d. EuroQoL visual analogue scale: https://euroqol.org/eq-5d-instruments/eq-5d-5l-about/
e. Short-Form 12: https://www.rand.org/health-care/surveys_tools/mos/12-item-short-form.html
f. Rotterdam Symptoms Check List questionnaire (RSCL): https://meetinstrumentenzorg.nl/wp-content/uploads/instrumenten/RSCL-handl.pdf

S6. Additional reported outcomes

Author	Diverting stomy AL vs non-AL (%)	P value	Neoadjuvant therapy AL vs non-AL (%)	P value	Tumor location AL vs non AL (mean distance from AV; cm)	P value	Surgical procedures AL vs non-AL	P value	Type of reinterventions AL vs non-AL	P value	Stoma status AL vs no AL (%)	P value	Onco. outcomes
**Arron et al.**	17 (27) : 223 (20)	0.19	14 (22) : 187 (17) (chemo)radiotherapy	0.23	NA	/	See table 2.	0.02	Radiological 4 (6) : 7 (12), Laparoscopic 11 (18): 10 (17), Open procedure 45 (71) : 35 (60), Other 3 (5) : 6 (10)	0.41	- 6 months 34 (72) : 108 (11)<br>- 12 months 15 (40) : 31 (4)	<0.01<br><0.01	NA
**Ashburn et al.**	27 (52) : 216 (27)	0.01	23 (67.6) : 206 (55.5)	0.4	9.7: 13.6	**0.001**	NA	/	NA	/	NA	/	NA
**di Cristofaro et al.**	NA	/	NA	/	NA	/	NA	/	NA	/	NA	/	

**Hain et al.**	Everyone	/	35 (76) : 61 (69) radiotherapy	0.36*	5.4 : 6.1	0.33*	Intersphincteric resection 17 (37) : 30 (34)	0.71*	NA	/	NA	NA	NA
**van Kooten et al.**	NA	/	NA	/	NA	/	NA	/	NA	/	Not AL specific	/	NA
**Lim et al.**	NA	/	Comparison between SCL and CL chemotherapy and radiotherapy	NS	NA	/	NA	/	NA	/	6 CL : 0 SCL	/	NA
**Marinatou et al.**	13 (52) : 18 (36)	0.28	12 (48) : 38 (76) chemo±radiotherapy	0.03	5.3 : 5.7	0.5	NA	/	NA	/	- 3 months 21 (84) : 13 (26)<br>- 6 months 20 (80) : 12 (24),<br>- 12 months 13 (52) : 1 (2)	0.0001<br>0.0001<br>0.0001	NA
**McGiffin et al.**	Exclusion criterium	/	NA	/	10 : 12	**0.011**	PME 5 (21) : 123 (61.5);<br>TME 19 (79) : 77 (38.5)	**<0.001**	NA	/	NA	/	NA
**Miura et al.**	Exclusion criterium	/	NA	/	NA	/	NA	/	NA	/	Permanent stoma formation rate was 16.7% for AL	/	NA
**Mongin et al.**	NA	/	17 (81) : 26 (62) radiotherapy	0.158	NA	/	Intersphincteric resection 10 (48) : 19 (45)	0.858	NA	/	all patients who still had a stoma after AL were excluded	/	NA

**di Re et al.**	24 (39.3) : 23 (37.7)	0.900	14 (23.0) : 14 (23.0) radiotherapy	> 0.999	- High 12 (19.7) : 14 (23.0)<br>- low 18 (29.5): 17 (27.9)<br>- ultra low 1 (50.8) : 30 (49.2)	0.900	NA	/	NA	/	NA	/	Disease free survival at 1, 3 and 5 years NS
**Riss et al.**	12 (75) : 8 (67)	0.0455	11 (68.8) : 9 (56.3) (chemo)radiotherapy	NS	NA	/	Same disctribution of iersphincteric, Low anterior and complete	NS	NA	/	NA	/	NA
**Westerduin et al.**	37 (71) : 82 (69)	0.83	29 (56) : 55 (47) chemotherapy<br>43 (83) : 68 (58) radiotherapy	0.27<br>0.001	NA	/	NA	/	NA	/	Temporary stoma formation in 87% of AL group	/	NA

*AL, anastomotic leakage; SCL, subclinical leak; CL, clinical leak; NS, not significant. *also not significant when subgroup analysis for symptomatic and non-symptomatic AL.*
